# Real-world experience with calcitonin gene-related peptide-targeted antibodies for migraine prevention: a retrospective observational cohort study at two Japanese headache centers

**DOI:** 10.1186/s12883-023-03521-y

**Published:** 2024-01-18

**Authors:** Mamoru Shibata, Kazuki Fujita, Eri Hoshino, Kazushi Minami, Kenzo Koizumi, Satoshi Okada, Fumihiko Sakai

**Affiliations:** 1 Department of Neurology, Tokyo Dental College Ichikawa General Hospital, 5-11-13 Sugano, Ichikawa, Chiba, 272-8513 Japan; 2 Saitama International Headache Center, Saitama Neuropsychiatric Institute, Saitama, Japan; 3 Saitama International Headache Center, Saitama, Japan

**Keywords:** Migraine, Calcitonin gene-related peptide (CGRP), Monoclonal antibody, Real world, Headache Impact Test-6 (HIT-6), Japanese

## Abstract

**Background:**

Although randomized controlled trials (RCTs) have shown that calcitonin gene-related peptide (CGRP)-targeted monoclonal antibodies (CGRP mAbs) are an efficacious and safe therapeutic modality for migraine prevention, their clinical benefits have not been well validated in Japanese patients in the real-world setting. The present study aimed to evaluate the real-world efficacy and safety of galcanezumab, fremanezumab, and erenumab in Japanese patients with migraine.

**Methods:**

This observational retrospective cohort study was conducted at two headache centers in Japan. Patients with migraine who had experienced treatment failure with at least one traditional oral migraine preventive agent were treated with a CGRP mAb de novo. The primary efficacy endpoints were the changes from baseline in monthly migraine days (MMDs) and Headache Impact Test-6 (HIT-6) score after 3 dosing intervals (V3). We explored whether demographic and clinical characteristics predicted therapeutic outcomes at V3.

**Results:**

Sixty-eight patients who completed three doses of a CGRP mAb (85.3% female [58/68], mean age: 46.2 ± 13.1 years) were included in the analysis. There were 19 patients with chronic migraine. The baseline MMDs were 13.4 ± 6.0. After 3 doses, the MMDs significantly decreased to 7.4 ± 5.5 (*p* < 0.0001), and the 50% response rate was 50.0%. HIT-6 score was significantly reduced from 66.7 ± 5.4 to 56.2 ± 8.7 after 3 doses (*P* = 0.0001). There was a positive correlation between the changes in MMDs and HIT-6 score from baseline after 2 doses (*p* = 0.0189). Those who achieved a ≥ 50% therapeutic response after the first and second doses were significantly more likely to do so at V3 (crude odds ratio: 3.474 [95% CI: 1.037 to 10.4], *p* = 0.0467). The most frequent adverse event was constipation (7.4%). None of the adverse events were serious, and there was no need for treatment discontinuation.

**Conclusions:**

This real-world study demonstrated that CGRP mAbs conferred Japanese patients with efficacious and safe migraine prevention, and an initial positive therapeutic response was predictive of subsequent favorable outcomes. Concomitant measurement of MMDs and HIT-6 score was useful in evaluating the efficacy of CGRP mAbs in migraine prevention.

**Supplementary Information:**

The online version contains supplementary material available at 10.1186/s12883-023-03521-y.

## Background

Migraine is a chronic disorder affecting more than one billion people worldwide [1]. This headache disorder is characterized by recurrent headache attacks of moderate to severe intensity, which interfere with daily activity. The interictal symptoms of migraine include allodynia, hypersensitivity, photophobia, phonophobia, osmophobia, visual/vestibular disturbances, and motion sickness [2]. The Eurolight project revealed that interictal symptoms, reported in 26.0% of patients with episodic migraine (EM), caused loss of productivity [3]. Hence, migraine causes considerable long-term disability in the sufferer’s daily and social life [3–5]. The Global Burden of Disease 2019 study showed that migraine is second among the world's causes of disability in terms of years lived with disability [6]. From a therapeutic viewpoint, effective and well-tolerated preventive therapy is key to enhancing the quality of life of migraineurs, especially those affected by high-frequency episodic migraine (HFEM) and chronic migraine (CM). CGRP plays a crucial role in migraine pathogenesis [7–9]. This neuropeptide is expressed in the trigeminal afferents innervating the dura, an important disease site associated with migraine [10, 11]. Monoclonal antibodies targeting either CGRP (galcanezumab, fremanezumab, and eptinezumab) or its receptor (erenumab) have been developed for migraine therapy. All of them have been efficacious in migraine prophylaxis, with favorable safety profiles in global RCTs [12–15]. Subsequent studies reported efficacy and safety for these monoclonal antibodies in migraineurs who had been unsuccessfully treated with preexisting preventive drugs, thus expanding the utility of the novel therapeutic agents [16–19]. However, it is important to note that migraine patients of Asian ethnicity were underrepresented in these studies. All four CGRP-targeted monoclonal antibodies are now approved for migraine prophylaxis. The strict inclusion and exclusion criteria imposed by the clinical trials limit the generalizability of the obtained results to patients seen in real-world situations.

RCTs have also demonstrated the efficacy and safety of galcanezumab [20], fremanezumab [21], and erenumab [22] for migraine prophylaxis in Japanese patients. Consequently, galcanezumab was first approved for migraine prevention in January 2021, followed by fremanezumab and erenumab in June 2021, in Japan. Although several Japanese single-center real-world studies have been published [23–26], there is still a paucity of real-world data on the efficacy and safety of CGRP mAbs in Japanese patients with migraine.

The purpose of the present study was to evaluate the effectiveness and safety of CGRP mAbs in real-world migraine therapy at two Japanese headache centers.

## Methods

### Study subjects

This is a retrospective, real-world study conducted at the Department of Neurology, Tokyo Dental College Ichikawa General Hospital and Saitama International Headache Center, Saitama Neuropsychiatric Institute, Saitama, Japan. It was approved by the Tokyo Dental College Ichikawa General Hospital Ethics Committee (Authorization number: I 23–02) and the Saitama Neuropsychiatric Institute Ethics Committee (SN I 23–002). We used opt-out procedures to obtain consent in the present study. The need for informed consent was waived by the Tokyo Dental College Ichikawa General Hospital Ethics Committee and the Saitama Neuropsychiatric Institute Ethics Committee, in accordance with national regulations (Ethical Guidelines for Medical and Biological Research Involving Human Subjects). All methods were carried out in accordance with relevant guidelines and regulations.

**Fig. 1:**
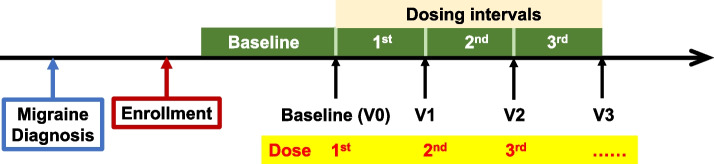
Study design. After confirming a diagnosis of migraine, consecutive eligible patients were enrolled. After the baseline period of more than 4 weeks, the enrolled patients were treated with 3 doses of one of the CGRP mAbs

We included migraine patients diagnosed by board-accredited neurologists according to the diagnostic criteria of the International Classification of Headache Disorders 3rd edition (ICHD-3) and treated de novo with one of the CGRP mAbs (galcanezumab, fremanezumab, and erenumab). All participants underwent a cranial MRI or CT scan to exclude secondary headache disorders. Blood tests were conducted as necessary. Before using a CGRP mAb, all the participants were required to have a history of treatment failure with at least one preexisting migraine prophylactic drug or traditional oral migraine preventive (TOMP) (lomerizine, propranolol, valproate, and amitriptyline) due to insufficient effectiveness, side effects, and/or poor tolerability. All patients had to have suffered from migraine attacks on at least 4 days per month on average during the last 3 months prior to their initial CGRP mAb treatment. Among the patients enrolled in this study, only those who completed 3 cycles of antibody administration and clinical assessment were eligible for data analysis (Fig. 1). Migraine cases complicated with medication-overuse headache (MOH) diagnosed in accordance with the ICHD-3 criteria were included. We excluded cases in which the baseline headache status was unclear.

### Migraine treatment with CGRP mAbs

CGRP mAb therapy was conducted as follows. For galcanezumab, a loading dose of 240 mg was administered subcutaneously for the first month, and 120 mg was administered monthly thereafter. For fremanezumab, all the analyzed patients received monthly subcutaneous administration at a dose of 225 mg. Erenumab was subcutaneously administered at a dose of 70 mg every 4 weeks. Concomitant use of TOMPs was allowed. No patients were treated with botulinum neurotoxin, which is not approved for chronic migraine in Japan. Triptans, acetaminophen, and NSAIDs were used for acute treatment as needed.

### Clinical evaluations and outcomes

Prior to the commencement of CGRP mAb treatment, we collected information about demographic characteristics, comorbidities, headache characteristics, baseline migraine days, accompanying symptoms, and acute headache medication use. The history of acute and preventive migraine therapy was reviewed. We instructed patients to fill out the headache diary every day to capture their headache status (duration, severity, and presence of accompanying symptoms). Patients were asked to complete the Headache Impact Test-6 (HIT-6), Generalized Anxiety Disorder-7 (GAD-7) and Patient Health Questionnaire-9 (PHQ-9).

In the present study, a migraine day was defined as any of the following:

- ➢ A calendar day (00:00 to 23:59) on which there were at least 2 consecutive hours of headache meeting the criteria for migraine with or without aura
- ➢ A calendar day (00:00 to 23:59) on which there were at least 2 consecutive hours of headache meeting the criteria for probable migraine, a migraine subtype where only one migraine criterion is missing.
- ➢ A calendar day (00:00 to 23:59) with headache of any duration that was treated with triptans.

We also verified the migrainous nature of each headache attack recorded in the headache diary by directly asking the patient. The first primary efficacy endpoint was the change from baseline in monthly migraine days (MMDs) during the third dosing interval. The number of MMDs was calculated per 28 days. The other primary efficacy endpoint was the change from baseline in the HIT-6 score during the third dosing interval. Moreover, 50%, 75% and 100% responder rates (RRs), defined as ≥ 50%, ≥ 75% and 100% reductions in migraine days, respectively, were calculated during the first three CGRP mAb dosing intervals. At every visit after the initiation of CGRP mAb treatment, patients were asked to report any adverse events. Among adverse events associated with CGRP mAbs, injection site reactions are known to be common [12–15]. Hence, only skin changes (erythema, swelling, eruption, etc.) lasting beyond the day of administration were regarded as adverse events. We considered a ≥ 20 mmHg increase in systolic blood pressure a significant blood pressure elevation.

### Statistical analysis

Statistical analyses were conducted with GraphPad Prism 8 (GraphPad Software, San Diego, CA, USA) and IBM SPSS Statistics ver. 29 (Armonk, NY, USA). Numerical data are expressed as mean with standard deviation (SD) or as 95% confidence interval (CI). Frequency data analyses were performed using the chi-square test or Fisher’s exact test. For numerical data, between-group comparisons were performed using analysis of variance (ANOVA) or the Kruskal–Wallis test, in accordance with the results of the D'Agostino-Pearson test for the normality of the data distribution. Multiple comparisons were carried out with Dunnett’s post hoc test or Dunn’s post hoc test. Two-way ANOVA was performed to compare the effects of the CGRP mAbs on the temporal trajectories of MMD and the HIT-6 score. For two-group comparisons, statistical analyses were conducted using Student’s t test or the Mann–Whitney test in accordance with the results of the D'Agostino-Pearson test. The correlation between MMD and HIT-6 score was evaluated as the Spearman rank-order correlation coefficient. The predictive ability of demographic and clinical parameters for 50% RR at visit 3 was analyzed by univariate and multivariate logistic regression models. No missing data were imputed. Statistical significance was set at *p* < 0.05 (two-tailed).

## Results

### Study participants

**Fig. 2:**
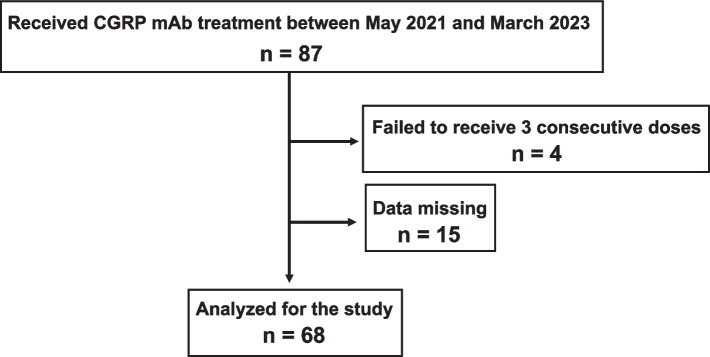
Enrollment and analysis of study participants

**Fig. 3:**
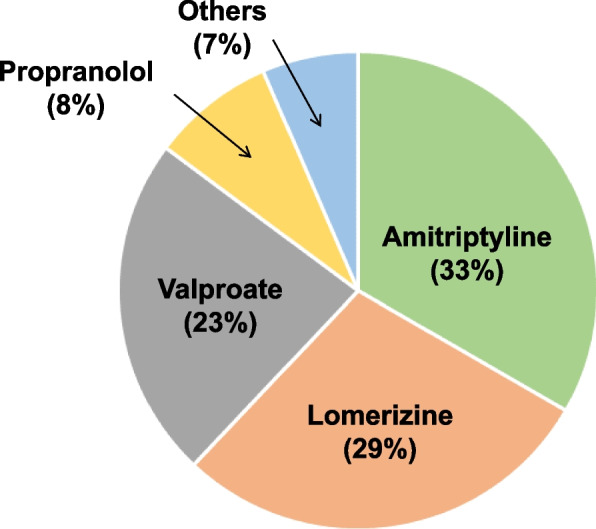
Previously used TOMPs by the study participants

From May 2021 through March 2023, 87 patients with migraine were treated de novo with one of the CGRP mAbs (Fig. 2). Of them, 83 patients completed 3 doses of CGRP mAbs (Fig. 2). We were not able to collect information about the baseline headache status in 15 patients, who were excluded from the study. Thus, 68 patients (30 patients at Tokyo Dental College Ichikawa General Hospital and 38 patients at Saitama Neuropsychiatric Institute) were eligible for data analysis (Fig. 2). The included patients (85.3% female [58/68], mean age: 46.2 ± 13.1 years) were of Japanese ethnicity. CM and medication overuse were found in 27.9% and 14.7% of patients, respectively. The demographic and baseline clinical characteristics of the analyzed participants are shown in Table 1. Galcanezumab, fremanezumab, and erenumab were administered in 31, 24, and 13 patients, respectively. There were no significant differences in demographic parameters among the treatment groups. Among the previously used TOMPs, amitriptyline was used by the most people (33%, Fig. 3). During the CGRP mAb dosing intervals, there was concomitant use of preexisting preventive drugs in 42 cases (61.8%, Table 1).

**Table 1:** Demographic and clinical characteristics of study participants

	**Whole Abs**	**Galcanezumab**	**Fremanezumab**	**Erenumab**	***p* value**
Number of patients	68	31	24	13	-
M/F	10/58	1/30	5/19	4/9	0.0839
Age, years	46.2 ± 13.1	43.8 ± 11.5	48.5 ± 15.6	47.9 ± 11.4	0.5733
Age of onset, years	24.6 ± 11.4	21.4 ± 8.5	29.4 ± 15.2	30.0 ± 11.7	0.0539
Disease duration, years	22.0 ± 11.1	22.2 ± 11.1	20.1 ± 10.0	25.3 ± 13.4	0.617
MO/MA	60/8	29/2	21/3	10/3	0.4829
Baseline MMDs	13.3 ± 6.0	14.0 ± 5.9	12.9 ± 5.5	12.9 ± 7.0	0.9108
CM diagnosis, n (%)	27.9	29.0	33.3	30.8	0.9818
Medication overuse, n (%)	14.7	19.4	4.7	7.7	0.361
Baseline HIT-6 score	66.7 ± 5.6	66.9 ± 5.9	67.2 ± 5.5	65.5 ± 5.8	0.8481
Number of previously used preventive drug classes, n (%)					
1	35	16	12	7	0.9827
2	20	8	8	4	0.9038
3	8	4	3	1	0.9779
≥ 4	3	3	0	0	0.3145
Comcomitant use of preventive drug(s)	42	18	16	8	0.9352
GAD-7 score^a^	6.9 ± 5.3	5.7 ± 4.7	7.8 ± 5.6	8.6 ± 6.5	0.7157
PHQ-9 score^a^	6.8 ± 5.3	4.7 ± 3.0	8.1 ± 5.6	10.0 ± 7.1	0.2413
Complications					
Hypertension, n (%)	11.8	16.1	8.3	7.7	0.7895
Diabetes mellitus, n (%)	1.5	0	14.7	0	0.6018
Dyslipidemia, n (%)	8.8	9.6	8.3	7.7	0.9965

^a^Data were obtained only in participants at Tokyo Dental College Ichikawa General Hospital

### Effects of CGRP mAbs on MMDs

**Fig. 4:**
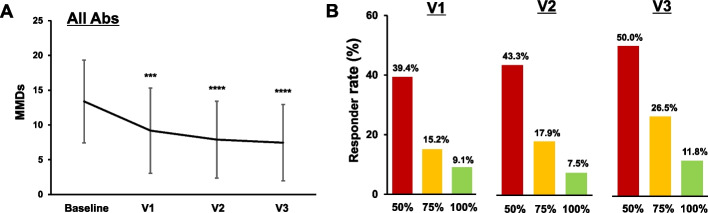
Effects of all the CGRP mAbs on MMDs. **A** Temporal profile of MMDs in patients treated with any CGRP mAb (*n* = 68). Data are shown as mean ± SD. Statistical analysis was performed using the Kruskal–Wallis test with Dunn’s post hoc test. ***p < 0.001, ****p < 0.0001. **B** 50%, 75%, and 100% RRs at V1, V2, and V3

We examined the effect of overall antibody treatment on the temporal changes in MMDs. The first, second, and third dosing intervals were 31.0 ± 5.3 days, 30.9 ± 4.0 days, and 31.4 ± 5.2 days, respectively. The baseline value of MMDs was 13.4 ± 6.0. After 3 doses, MMDs significantly decreased to 7.4 ± 5.5 (*p* < 0.0001, Fig. 4A). The 50% RR was 39.4%, 43.3% and 50.0% at V1, V2, and V3, respectively (Fig. 4B, red bars). Significant MMD reductions from baseline to V3 were observed in both CM (9.0 ± 6.0 vs. 19.1 ± 6,1, *p* = 0.0001, Supplementary Fig. 1A) and EM (6.8 ± 5.2 vs. 11.2 ± 4.2, *p* < 0.0001, Supplementary Fig. 1B). The RRs for EM and CM are shown in Supplementary Fig. 1C and D, respectively.

### Comparison of the therapeutic effects on MMDs among CGRP mAbs

**Fig. 5:**
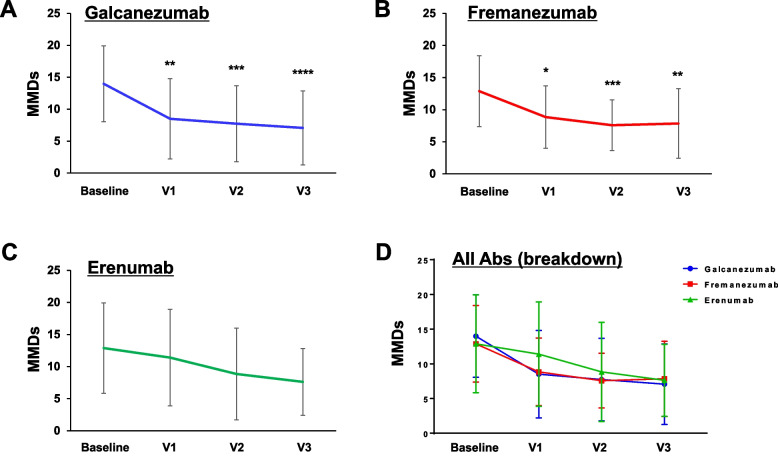
Effects of each CGRP mAb on MMDs. Temporal profiles of MMDs in patients treated with galcanezumab (*n* = 31, **A**), fremanezumab (*n* = 24, **B**), and erenumab (*n* = 13, **C**). Data are shown as mean ± SD. Statistical analysis was performed using one-way ANOVA with Dunnett’s post hoc test for galcanezumab and the Kruskal–Wallis test with Dunn’s post hoc test for fremanezumab and erenumab. *p < 0.05, **p < 0.01, ***p < 0.001, ****p < 0.0001. **D** Overlay line graph depicting the temporal profiles of each CGRP mAb

As shown in Fig. 5A–C, galcanezumab and fremanezumab significantly reduced MMDs after 3 doses (galcanezumab: 7.1 ± 5.8 vs. 14.0 ± 5.9, *p* = 0.0001; fremanezumab: 7.8 ± 5.4 vs. 12.1 ± 5.5, *p* = 0.0042), whereas there was no significant change in MMDs after 3 doses of erenumab (7.6 ± 5.2 vs. 12.9 ± 7.0, *p* = 0.1229). We next analyzed the effects of each CGRP mAb on MMDs using two-way ANOVA. There was a significant effect of time (F _(3, 257)_ = 11.74, *p* < 0.001), but there was no significant effect of CGRP mAb type (F _(2, 257)_ = 0.6174, *p* = 0.154) or the interaction between them (F _(6, 257)_ = 0.4534, *p* = 0.8423). There were no significant differences in MMDs among CGRP mAbs at any timepoint (Fig. 5D).

### Effects of CGRP mAb treatment on HIT-6 score

**Fig. 6:**
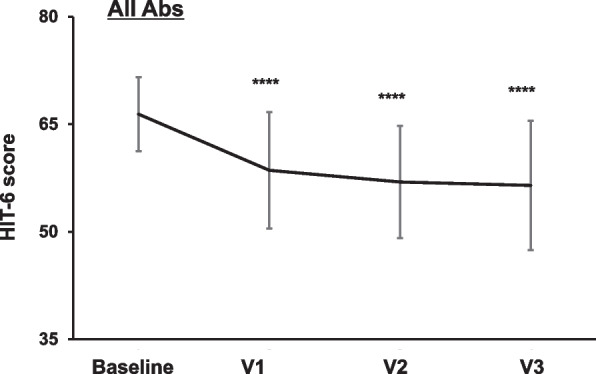
Effects of all the CGRP mAbs on HIT-6 score. Temporal profile of the HIT-6 score in patients treated with any CGRP mAb (*n* = 68). Data are shown as mean ± SD. Statistical analysis was performed using one-way ANOVA with Dunnett’s post hoc test. ****p < 0.0001

We investigated the effects of CGRP mAb treatment on the HIT-6 score. Considering CGRP mAb treatment overall, the baseline HIT-6 score was 66.7 ± 5.4, and after 3 doses, we observed a significant reduction to 56.2 ± 8.7 (*P* = 0.0001, Fig. 6). Significant improvements in the HIT-6 score at V3 were observed in both EM (56.8 ± 8.6 vs. 66.4 ± 5.8, *p* = 0.0001) and CM (54.9 ± 9.1 vs. 67.5 ± 5.2, *p* = 0.0001) (Supplementary Fig. 2).

### Comparison of the therapeutic effects on HIT-6 score among CGRP mAbs

**Fig. 7:**
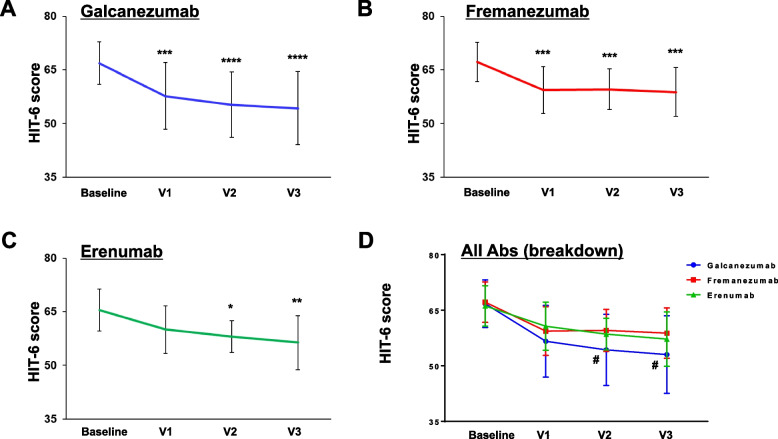
Effects of each CGRP mAb on HIT-6 score. Temporal profiles of HIT-6 score in patients treated with galcanezumab (*n* = 31, **A**), fremanezumab (*n* = 24, **B**), and erenumab (*n* = 13, **C**). Data are shown as mean ± SD. Statistical analysis was performed using one-way ANOVA with Dunnett’s post hoc test for galcanezumab and erenumab and the Kruskal–Wallis test with Dunn’s post hoc test for erenumab. *p < 0.05, **p < 0.01, ***p < 0.001, ****p < 0.0001. **D** Overlay line graph depicting the temporal profiles of each CGRP mAb. Between-group comparisons at each timepoint were performed with Dunnett’s post hoc test. #*p* < 0.05, galcanezumab vs. fremanezumab

All three CGRP mAbs significantly reduced the HIT-6 score after 3 doses (galcanezumab: 54.3 ± 10.2 vs. 66.9 ± 5.9, *p* = 0.0001; fremanezumab: 58.8 ± 6.8 vs. 67.2 ± 5.5, *p* = 0.0003; erenumab: 56.4 ± 7.5 vs. 65.5 ± 5.8, *p* = 0.0023, Fig. 7A). Two-way ANOVA revealed that there were significant effects of time (F _(3, 218)_ = 20.09, *p* < 0.0001) and CGRP mAb type (F _(2, 218)_ = 5.643, *p* = 0.0041), without an interaction between them (F _(6, 218)_ = 0.8083, *p* = 0.5644). Multiple comparisons detected significant differences between galcanezumab and fremanezumab at V2 (mean differences: -5.3 [95% CI: -10.2 to -0.3], *p* = 0.0338) and V3 (mean differences: -5.8 [95% CI: -11.0 to -0.7], *p* = 0.0242 (Fig. 7B).

### Temporal profiles of the distributions of MMDs and HIT-6 score

There was no correlation between MMDs and HIT-6 score at baseline (Supplementary Fig. 3A). We examined the correlations between the MMD difference from baseline and the HIT-6 score differences from baseline after the initiation of CGRP-targeted antibody treatment. The scatter plots showed that there was a tendency for the distribution to shift toward the lower left part (Supplementary Fig. 3B–D). There was a statistically significant correlation between the change in MMDs and the change in the HIT-6 score from baseline to V2 (*p* = 0.0189).

### Prediction of therapeutic response at V3

To explore demographic and clinical factors associated with the achievement of ≥ 50% therapeutic response at V3, age, disease duration, baseline MMDs, and baseline HIT-6 score were compared between the ≥ 50% responders and nonresponders at V3. There were no significant between-group differences in any of these factors (Supplementary Table 1). These factors were not found to be significant contributors to ≥ 50% therapeutic response at V3 by univariate or multivariate logistic regression analysis (Supplementary Table 2).

Complete data on MMDs at baseline and the first three visits were available for 65 participants (Supplementary Fig. 4). Based on their data, we asked whether the therapeutic response at V3 could be predicted from the response status at V1 or V2 by calculating positive and negative predictive values. The ≥ 50% responders at both V1 and V2 were significantly more likely to be ≥ 50% responders at V3 than nonresponders at both V1 and V2 were (crude odds ratio: 3.474 [95% CI: 1.037 to 10.4], *p* = 0.0467, Supplementary Table 3).

### Adverse events

The treatment-emergent adverse events reported during the study period are shown in Table 2. Constipation was the most frequent adverse event (7.4%). None of the adverse events were serious, and treatment discontinuation was not needed.

**Table 2:** Treatment-emergent adverse events

	**n (%)**
Total patients	**9 (13.2%)**
Injection-site reactions	**2 (2.9%)**
Constipation	**5 (7.4%)**
Blood pressure elevation (20 mmHg <)	**1 (1.5%)**
Alopecia	**1 (1.5%)**
Diarrhea	**1 (1.5%)**

## Discussion

Our two-center real-world study demonstrated that CGRP mAbs were effective in migraine prophylaxis in Japanese patients with a history of treatment failure with at least one TOMP in terms of the changes from baseline in MMDs and HIT-6 score. Significant reductions in MMDs were achieved by galcanezumab and fremanezumab. The HIT-6 score was significantly decreased by all CGRP mAbs. There was no significant correlation between MMDs and the HIT-6 score at baseline. After CGRP mAb treatment, a significant positive correlation between the changes in MMDs and HIT-6 score from baseline was observed at V2. For achieving a ≥ 50% reduction in MMDs at V3, the positive therapeutic response at V1 and V2 was found to be a significant predictor. With respect to safety, there were no serious treatment-emergent adverse events. Taken together, our real-world data confirm that CGRP mAbs provide excellent migraine prophylaxis with favorable safety and tolerability profiles in Japanese patients with migraine. Unlike previous Japanese real-world studies [23–26], the present study was conducted at two independent institutions, extending the generalizability of the data.

The present study evaluated the temporal changes in MMDs for each CGRP mAb. Unlike galcanezumab and fremanezumab, erenumab did not significantly decrease MMDs from baseline at V3. However, we must interpret our findings cautiously due to the low number of patients treated with erenumab. With more patients, the results with erenumab would also have been statistically significant. The comparison of efficacy in reducing MMDs among CGRP mAbs is a clinically relevant topic, which should be explored with a much larger population [25, 27–29].

All the CGRP mAbs used in the present study improved the HIT-6 score from baseline at V3. HIT-6 score is a patient-reported outcome measure reflecting the negative impact of migraine attacks on normal daily activity [30]. Hence, the present study provides evidence that CGRP mAbs abate migraine-associated disability, in line with previous reports [31–35]. In our data, there was no significant correlation between MMDs and HIT-6 scores in the study subjects at baseline, implying that migraine-associated disability was not determined simply by the number of MMDs. Other factors, such as headache intensity and duration of each migraine attack, contribute to the disability as well. Although both MMDs and HIT-6 score declined after CGRP mAb treatment, we found a significant correlation between the MMD difference from baseline and the HIT-6 score difference from baseline only at V2. HIT-6 scoring is useful in assessing the effect of migraine-associated disability factors other than MMDs. The number of MMDs alone does not seem to be sufficient to encompass the complexity of therapeutic benefits of CGPP Abs. Our data highlight the utility of implementing HIT-6 scoring along with MMD count to thoroughly evaluate the disease condition of migraineurs. To the best of our knowledge, this is the first real-world study to examine the effects of CGRP mAbs on both MMDs and HIT-6 score in Japanese patients with migraine.

The 50% RR is considered a useful index to compare the efficacy of prophylactic therapy for migraine among different studies. In RCTs, galcanezumab, fremanezumab, and erenumab yielded 50% RRs at 3 months in 39.7–62.3% of patients with EM and 27.5–50.0% of patients with CM [12–14, 36–40]. Real-world studies evaluating these CGRP mAbs for HFEM and CM reported 3-month 50% RRs of 39.5–61.5% [23, 24, 31, 34, 41, 42]. Hence, the results of our study are consistent with these previous findings. Our analysis revealed that the accomplishment of 50% RR at V1 and V2 increased the likelihood of a positive outcome at V3. This finding supports the therapeutic consistency of CGRP mAbs [26, 34, 35, 41, 43, 44].

Attempts have been made to detect predictive factors for a positive therapeutic response to CGRP mAbs in the real-world setting [45]. We explored whether demographic and clinical parameters could predict the therapeutic response at V3. However, age, disease duration, baseline MMDs, and baseline HIT-6 were not relevant to 50% RR at V3. Although previous studies found these clinical parameters to be predictors of clinical efficacy, it should be pointed out that there are clear discrepancies among such data in terms of age [24, 31, 46] and baseline MMDs [25, 46, 47]. Hence, there is the possibility that the effectiveness of these parameters as therapeutic predictors is influenced by the difference in study populations.

**Fig. 8:**
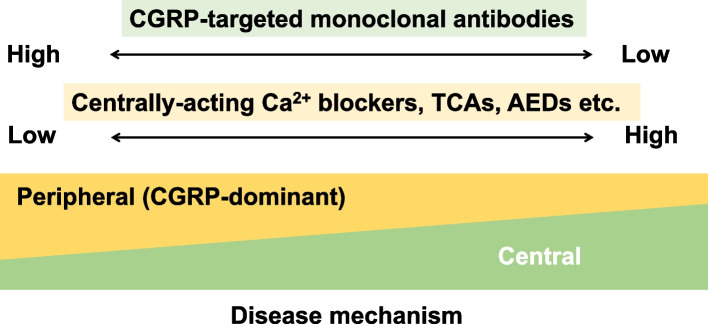
Schematic representation depicting a possible favorable effect of combinatorial treatment with CGRP mAbs, which mainly act mainly at peripheral sites, and centrally acting preventive drugs, such as Ca^2+^ blockers, tricyclic antidepressants (TCAs), and antiepileptic drugs (AEDs)

This was a real-world study in which the use of other prophylactic drugs was not restricted, so 61.8% of participants were concomitantly taking at least one TOMP. Many TOMPs can act in the central nervous system [48]. Amitriptyline and lomerizine were often used in our study subjects. Currently, lomerizine is used exclusively in Japan. This agent is a centrally acting calcium channel blocker similar to flunarizine, a globally used migraine preventive drug [49–51]. Migraine is a complex disorder involving the central nervous system as well as the trigeminovascular system [1, 7, 8]. CGRP mAbs are likely to rectify abnormalities in the trigeminovascular system [9]. Hence, the combination of CGRP mAbs and centrally acting migraine prophylaxis may confer a potent therapeutic effect by affecting complementary pathways (Fig. 8), although there is still insufficient evidence to support this paradigm [52].

There are limitations to the present study. First, the relatively small number of study subjects and inequality of assignment to each CGRP mAb lowered the statistical power. Because the clinical data were not collected in a prospective manner, 15 cases (18%) were excluded due to missing baseline data. In Japan, galcanezumab was approved earliest, so more patients were treated with this antibody than any other. Second, in accordance with the general Japanese insurance policy, patients must cover 30% of their drug costs. Hence, we are able to apply CGRP mAb therapy only to those who can afford it, generating a selection bias stemming from the economic status of patients. Third, we did not collect detailed information about the clinical features of migraine attacks, such as the nature of the headaches and their laterality and the presence of accompanying symptoms. Unilaterality, pulsatile nature, vomiting, cranial autonomic symptoms, allodynia, osmophobia, and good response to triptans have been identified as predictive factors for therapeutic response [25, 43, 53, 54]. Conversely, concomitant depression and obesity are known to predict poor outcomes [43, 53]. Hence, our failure to collect detailed clinical information may have led to our inability to find effective predictors for therapeutic response at V3. Lastly, the short follow-up duration may have limited our study, because it has been pointed out that the effectiveness of CGRP mAbs might be evident up to 6 months of consecutive treatment [52].

## Conclusions

The present study provides new real-world evidence of the efficacy and safety of CGRP mAbs in Japanese migraine sufferers. In particular, our data first demonstrated the utility of concomitant monitoring of MMDs and the HIT-6 score to evaluate the efficacy of CGRP mAbs in Japanese patients. In addition, each CGRP mAb exhibited distinct improving actions on MMDs and the HIT-6 score.

### Supplementary Information

**Additional file 1: Supplementary Figure 1.** Effects of all the CGRP mAbs on MMDs.

**Additional file 2: Supplementary Figure 2.** Effects of all the CGRP mAbs on HIT-6 score in the EM and CM subgroups.

**Additional file 3: Supplementary Figure 3.** Temporal profiles of the distributions of MMDs and HIT-6 score.

**Additional file 4: Supplementary Figure 4.** Tree diagram depicting the numbers of ≥50% responders (green lines) and nonresponders (red lines) at each visit.

**Additional file 5: Supplementary Table 1.** Comparison of demographic and clinical characteristics between ≥50% responders and non-responders.

**Additional file 6: Supplementary Table 2.** Logistic regression analysis for 50% RR at V3.

**Additional file 7: Supplementary Table 3.** Prediction of 50% response at V3 from the response status at V1 and V2.

**Additional file 8: Supplementary file 8.** Visual abstract of the present study.

## Data Availability

Not applicable.

## Abbreviations

AED: Antiepileptic drug
CGRP: Calcitonin gene-related peptide
CI: Confidence interval
CM: Chronic migraine
EM: Episodic migraine
GAD-7: General Anxiety Disorder-7
HFEM: High-frequency episodic migraine
HIT-6: Headache Impact Test-6
ICHD-3: International Classification of Headache Disorders 3rd edition
MDAS: Migraine Disability Assessment Test
MMD: Monthly migraine day
MOH: Medication-overuse headache
OR: Odds ratio
PHQ-9: Patient Health Questionnaire-9
RCT: Randomized clinical trial
RR: Responder rate
SD: Standard deviation
