# Correction: Real‑world experience with calcitonin gene‑related peptide‑targeted antibodies for migraine prevention: a retrospective observational cohort study at two Japanese headache centers

**DOI:** 10.1186/s12883-024-03665-5

**Published:** 2024-05-09

**Authors:** Mamoru Shibata, Kazuki Fujita, Eri Hoshino, Kazushi Minami, Kenzo Koizumi, Satoshi Okada, Fumihiko Sakai

**Affiliations:** 1 Department of Neurology, Tokyo Dental College Ichikawa General Hospital, 5‑11‑13 Sugano, Ichikawa, Chiba 272‑8513 Japan; 2 Saitama International Headache Center, Saitama Neuropsychiatric Institute, Saitama, Japan; 3 Saitama International Headache Center, Saitama, Japan

**Correction: BMC Neurol 24, 32 (2024)**

10.1186/s12883-023-03521-y

Following publication of the original article [1], the authors would like to correct a couple of errors in numerical data under the headings “Effects of CGRP mAbs on MMDs”, “Comparison of the therapeutic effects on MMDs among CGRP mAbs” and Table 1.

**Effects of CGRP mAbs on MMDs**.

The sentence currently reads:

Significant MMD reductions from baseline to V3 were observed in both **EM** (9.0 ± **7.3** vs. 19.1 ± 6,1, *p* = 0.0001, Supplementary Fig. 1A) and **CM** (**7.1 ± 4.5** vs. 11.2 ± 4.2, *p* < 0.0001, Supplementary Fig. 1B).

The sentence should read:

Significant MMD reductions from baseline to V3 were observed in both **CM** (9.0 ± **6.0** vs. 19.1 ± 6,1, *p* = 0.0001, Supplementary Fig. 1A) and **EM** (**6.8 ± 5.2** vs. 11.2 ± 4.2, *p* < 0.0001, Supplementary Fig. 1B).

**Comparison of the therapeutic effects on MMDs among CGRP mAbs**.

The sentence currently reads:

As shown in Fig. 5A–C, galcanezumab and fremanezumab significantly reduced MMDs after 3 doses (galcanezumab: 7.1 ± 5.8 vs. 14.0 ± 5.9, *p* = 0.0001; fremanezumab: 7.8 ± 5.4 vs. **19.1** ± 5.5, *p* = 0.0042),…

The sentence should read:

As shown in Fig. 5A–C, galcanezumab and fremanezumab significantly reduced MMDs after 3 doses (galcanezumab: 7.1 ± 5.8 vs. 14.0 ± 5.9, *p* = 0.0001; fremanezumab: 7.8 ± 5.4 vs. **12.1** ± 5.5, *p* = 0.0042),….

In **Table 1** under column Fremanezumab, 67.2 ± **2 5.5** should be changed to 67.2 ± **5.5**.

The original article [1] has been updated.
