# Supplementary material for: Real-world experience with calcitonin gene-related peptide-targeted antibodies for migraine prevention: a retrospective observational cohort study at two Japanese headache centers
Source: BMC Neurol. 2024 Jan 18;24:32. doi: 10.1186/s12883-023-03521-y (PMC10795407; doi:10.1186/s12883-023-03521-y)
Supplement: Supplementary file 1 — Additional file 1: Supplementary Figure 1. Effects of all the CGRP mAbs on MMDs. [file 12883_2023_3521_MOESM1_ESM.pdf]

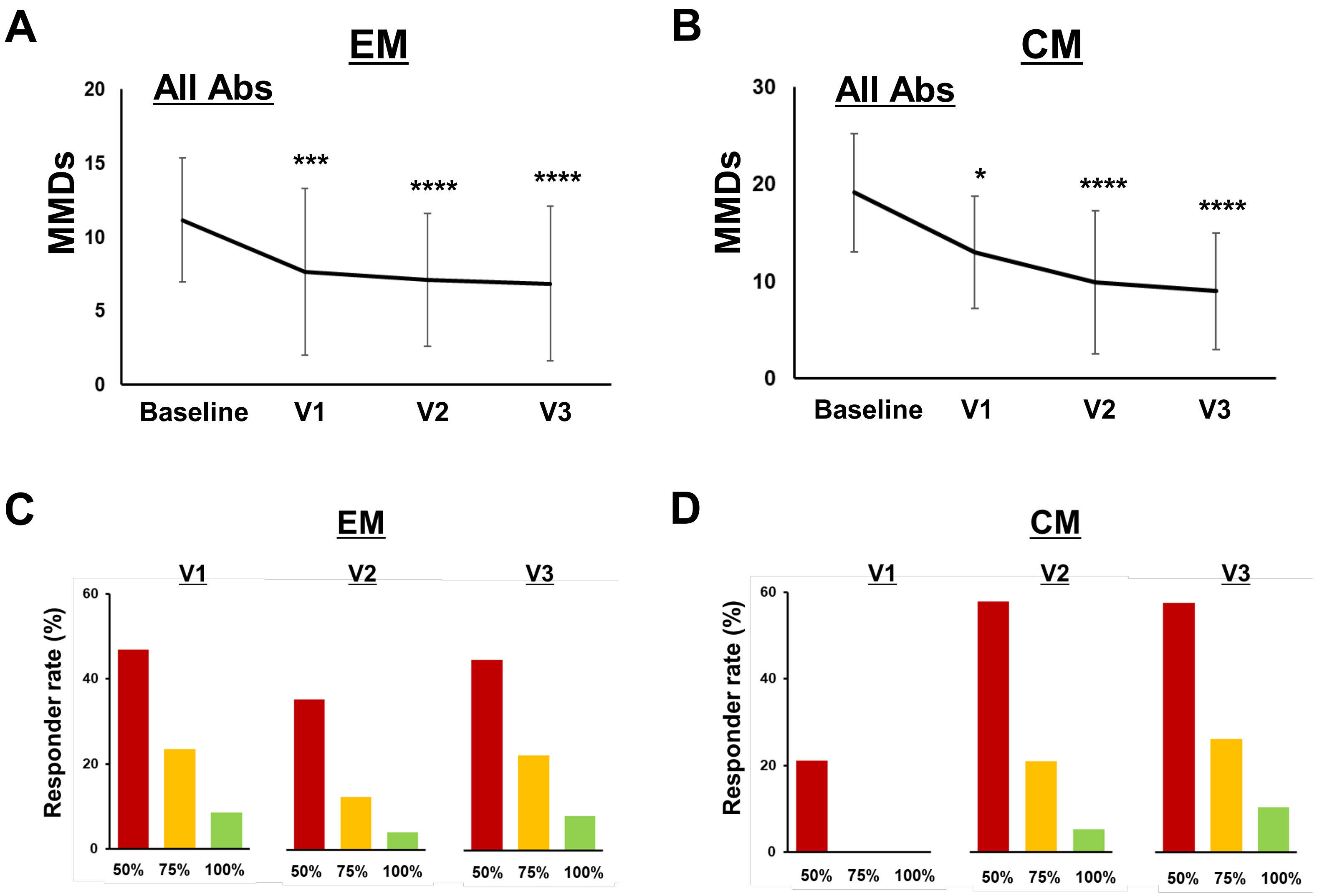

Additional file 1. Supplementary Figure 1

Effects of all the CGRP mAbs on MMDs. (A) Temporal profile of MMDs in patients with EM treated with any CGRP mAb (n = 49). Data are shown as mean ± SD. Statistical analysis was performed using the Kruskal–Wallis test with Dunn's post hoc test. (B) Temporal profile of MMDs in patients with CM treated with any CGRP mAb (n = 19). Data are shown as mean ± SD. Statistical analysis was performed by one-way ANOVA with Dunnett's post hoc test. *p < 0.05, ***p < 0.001, ****p < 0.0001. (C) 50%, 75%, and 100% RRs at V1, V2, and V3 in patients with EM. (D) 50%, 75%, and 100% RRs at V1, V2, and V3 in patients with CM.
