# Supplementary material for: Real-world experience with calcitonin gene-related peptide-targeted antibodies for migraine prevention: a retrospective observational cohort study at two Japanese headache centers
Source: BMC Neurol. 2024 Jan 18;24:32. doi: 10.1186/s12883-023-03521-y (PMC10795407; doi:10.1186/s12883-023-03521-y)
Supplement: Supplementary file 2 — Additional file 2: Supplementary Figure 2. Effects of all the CGRP mAbs on HIT-6 score in the EM and CM subgroups. [file 12883_2023_3521_MOESM2_ESM.pdf]

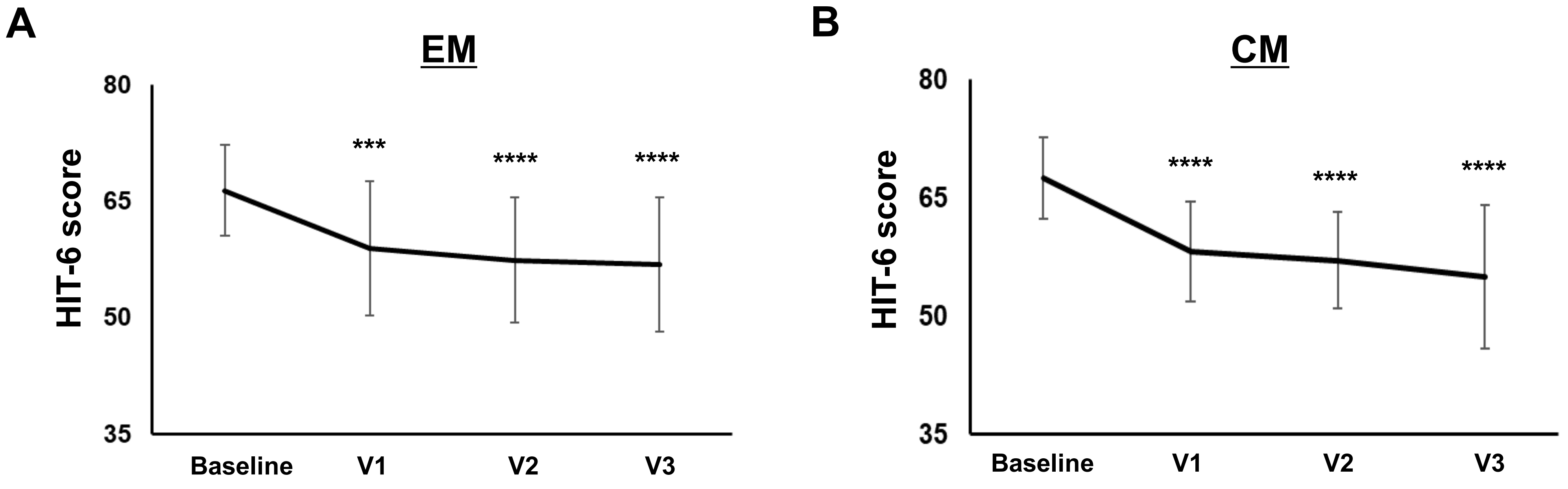

Additional File 2. Supplementary Figure 2

Effects of all the CGRP mAbs on HIT-6 score in the EM and CM subgroups. (A) Temporal profile of HIT-6 score in patients with EM treated with any CGRP mAb (N = 49). (B) Temporal profile of HIT-6 score in patients with CM treated with any CGRP mAb (N = 19). Data are shown as mean ± SD. Statistical analysis was performed with one-way ANOVA with Dunnett's post hoc test. ***$p < 0.001$, ****$p < 0.0001$.
