# Supplementary material for: Real-world experience with calcitonin gene-related peptide-targeted antibodies for migraine prevention: a retrospective observational cohort study at two Japanese headache centers
Source: BMC Neurol. 2024 Jan 18;24:32. doi: 10.1186/s12883-023-03521-y (PMC10795407; doi:10.1186/s12883-023-03521-y)
Supplement: Supplementary file 3 — Additional file 3: Supplementary Figure 3. Temporal profiles of the distributions of MMDs and HIT-6 score. [file 12883_2023_3521_MOESM3_ESM.pdf]

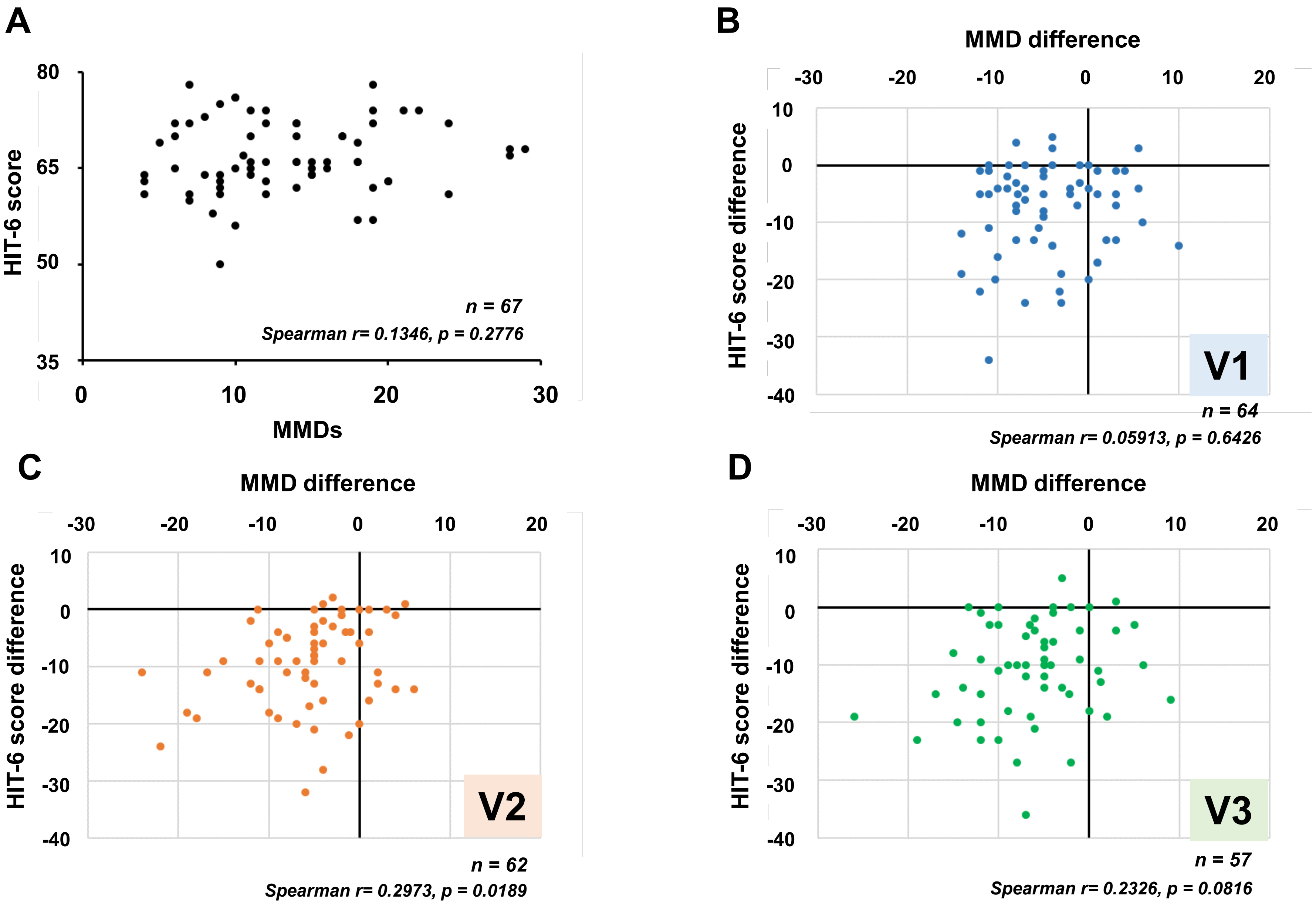

Additional file 3. Supplementary Fig. 3. Temporal profiles of the distributions of MMDs and HIT-6 score. (A) Scatter plots of MMDs and HIT-6 score at baseline. (B–D) Scatter plots of the MMD difference from baseline and the HIT-6 score difference from baseline at V1 (B), V2 (C), and V3 (D). Correlations were the Spearman rank-order correlation coefficient.
