# Supplementary material for: Real-world experience with calcitonin gene-related peptide-targeted antibodies for migraine prevention: a retrospective observational cohort study at two Japanese headache centers
Source: BMC Neurol. 2024 Jan 18;24:32. doi: 10.1186/s12883-023-03521-y (PMC10795407; doi:10.1186/s12883-023-03521-y)
Supplement: Supplementary file 4 — Additional file 4: Supplementary Figure 4. Tree diagram depicting the numbers of ≥50% responders (green lines) and nonresponders (red lines) at each visit. [file 12883_2023_3521_MOESM4_ESM.pdf]

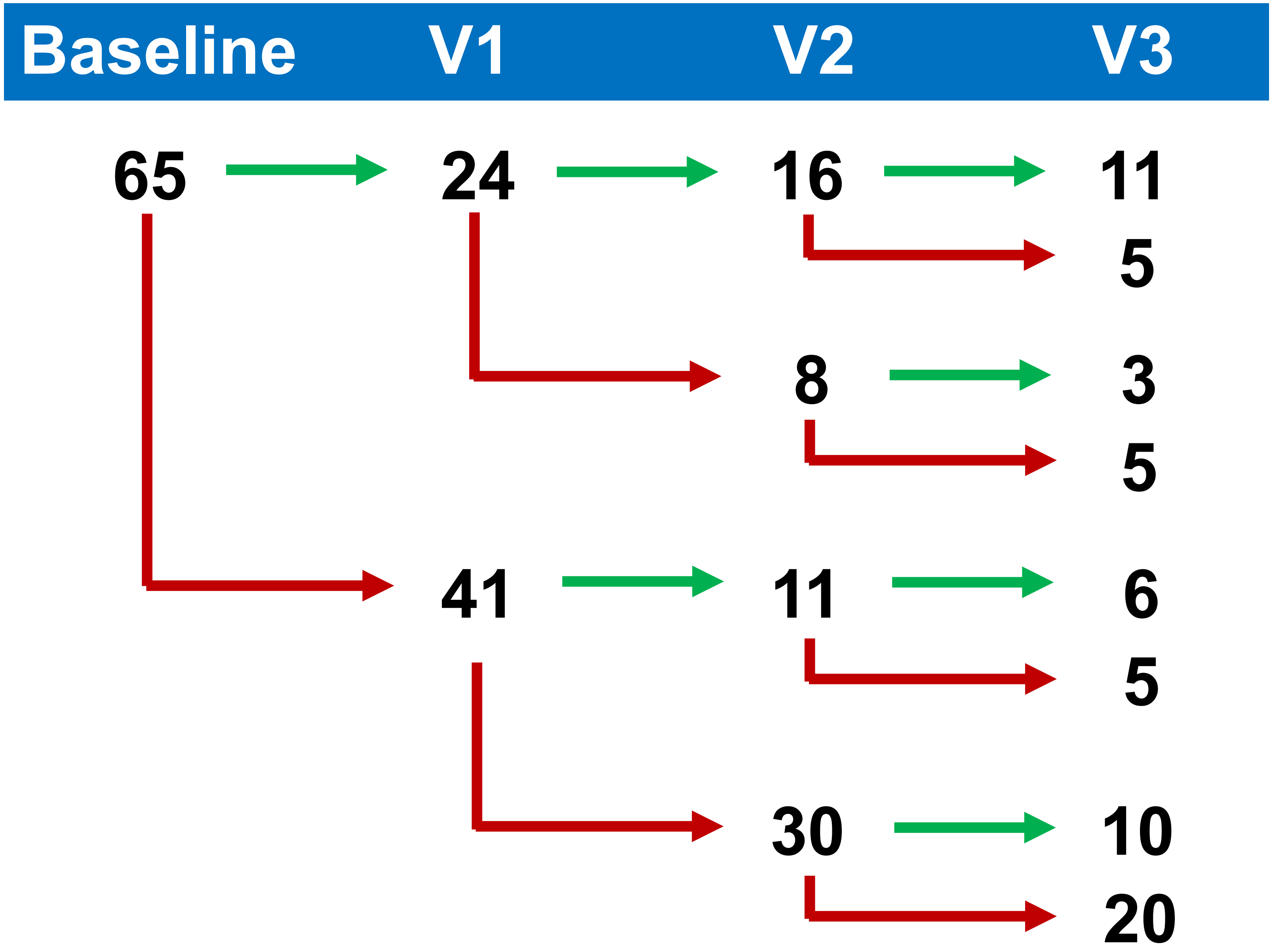

Additional file 4. Supplementary Fig. 4.
Tree diagram depicting the numbers of ≥50% responders (green lines) and nonresponders (red lines) at each visit.
