# Supplementary material for: Real-world experience with calcitonin gene-related peptide-targeted antibodies for migraine prevention: a retrospective observational cohort study at two Japanese headache centers
Source: BMC Neurol. 2024 Jan 18;24:32. doi: 10.1186/s12883-023-03521-y (PMC10795407; doi:10.1186/s12883-023-03521-y)
Supplement: Supplementary file 5 — Additional file 5: Supplementary Table 1. Comparison of demographic and clinical characteristics between ≥50% responders and non-responders. [file 12883_2023_3521_MOESM5_ESM.pdf]

Supplementary file 5. Supplementary Table 1. Comparison of demographic and clinical characteristics between ≥50% responders and non-responders.

	50% RR at V3		p value
	Responder (N = 31)	Non-responder (N = 37)	
**Age**	46.7 ± 14.3	45.9 ± 12.2	0.822
**Disease duration**	22.1 ± 12.8	22.2 ± 9.9	0.965
**Age of onset**	25.5 ± 11.8	24.0 ± 11.1	0.606
**Baseline MMDs**	14.5 ± 6.3	12.5 ± 5.6	0.177
**Baseline HIT-6 score**	66.7 ± 5.4	65.7 ± 5.6	0.457
