# Supplementary material for: Real-world experience with calcitonin gene-related peptide-targeted antibodies for migraine prevention: a retrospective observational cohort study at two Japanese headache centers
Source: BMC Neurol. 2024 Jan 18;24:32. doi: 10.1186/s12883-023-03521-y (PMC10795407; doi:10.1186/s12883-023-03521-y)
Supplement: Supplementary file 6 — Additional file 6: Supplementary Table 2. Logistic regression analysis for 50% RR at V3. [file 12883_2023_3521_MOESM6_ESM.pdf]

Supplementary file 6. Supplementary Table 2. Logistic regression analysis for 50% RR at V3

	**Standardized regression coefficient**	**SE**	**Wald**	**OR (95% CI)**	**p value**
**Univariate**					
**Age**	0.004	0.019	0.053	1.004 (0.968 to 1.042)	0.818
**Disease duration**	-0.001	0.023	0.002	0.999 (0.956 to 1.044)	0.964
**Baseline MMDs**	0.057	0.043	1.816	1.059 (0.974 to 1.151)	0.178
**Baseline HIT-6 score**	0.034	0.566	1.035	1.035 (0.947 to 1.130)	0.452
**Multivariate**					
**Age**	0.012	0.025	0.245	1.012 (0.964 to 1.063)	0.621
**Disease duration**	0.005	0.027	0.033	1.005 (0.952 to 1.061)	0.855
**Baseline MMDs**	0.050	0.044	1.298	1.051 (0.965 to 1.146)	0.255
**Baseline HIT-6 score**	0.039	0.048	0.666	1.040 (0.947 to 1.142)	0.414
