# Supplementary material for: Real-world experience with calcitonin gene-related peptide-targeted antibodies for migraine prevention: a retrospective observational cohort study at two Japanese headache centers
Source: BMC Neurol. 2024 Jan 18;24:32. doi: 10.1186/s12883-023-03521-y (PMC10795407; doi:10.1186/s12883-023-03521-y)
Supplement: Supplementary file 7 — Additional file 7: Supplementary Table 3. Prediction of 50% response at V3 from the response status at V1 and V2. [file 12883_2023_3521_MOESM7_ESM.pdf]

Supplementary file 7. Supplementary Table 3. Prediction of 50% response at V3 from the response status at V1 and V2.

	**≥50% Predicted at V3**		**cOR**	**95% CI**	**p value**
	**Positive predictive value**	**Negative predictive value**			
**≥50% Both V1 and V2**	0.6875	0.6122	3.474	1.037–10.4	0.0467
**≥50% Either V1 or V2**	0.4737	0.5435	1.071	0.3579–3.101	0.8995
**≥50% Neither V1 nor V2**	0.3333	0.4286	0.375	0.1385–1.038	0.0549

cOR: crude odds ratio
